# A Self-Adaptive Discrete PSO Algorithm with Heterogeneous Parameter Values for Dynamic TSP

**DOI:** 10.3390/e21080738

**Published:** 2019-07-27

**Authors:** Łukasz Strąk, Rafał Skinderowicz, Urszula Boryczka, Arkadiusz Nowakowski

**Affiliations:** Institute of Computer Science, University of Silesia in Katowice, Będzińska 39, 41-205 Sosnowiec, Poland

**Keywords:** dynamic traveling salesman problem, pheromone, discrete particle swarm optimization, heterogeneous, homogeneous

## Abstract

This paper presents a discrete particle swarm optimization (DPSO) algorithm with heterogeneous (non-uniform) parameter values for solving the dynamic traveling salesman problem (DTSP). The DTSP can be modeled as a sequence of static sub-problems, each of which is an instance of the TSP. In the proposed DPSO algorithm, the information gathered while solving a sub-problem is retained in the form of a pheromone matrix and used by the algorithm while solving the next sub-problem. We present a method for automatically setting the values of the key DPSO parameters (except for the parameters directly related to the computation time and size of a problem).We show that the diversity of parameters values has a positive effect on the quality of the generated results. Furthermore, the population in the proposed algorithm has a higher level of entropy. We compare the performance of the proposed heterogeneous DPSO with two ant colony optimization (ACO) algorithms. The proposed algorithm outperforms the base DPSO and is competitive with the ACO.

## 1. Introduction

In recent years, considerable attention has been paid to optimization in a dynamically-changing environment in which the problem being solved is modified periodically or even continuously [1]. This interest is related to the growing practical demand for such solutions. For example, in the problem of task scheduling in a factory, a change to the production schedule might be required if there is a malfunction of part of the production line. The optimization algorithms should be able to adapt rapidly to changes so that the quality of the generated solutions remains acceptable. A problem in which the input data are conditional upon time is called a dynamic optimization problem (DOP). The aim of optimization in a DOP is a constant trace and an adaptation to changes, in order to allow high-quality solutions to be found efficiently [2].

**Figure 1:**
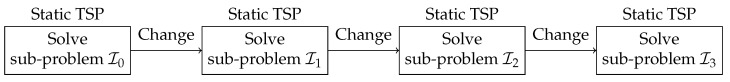
An example of a dynamic optimization problem (DOP), the DTSP. A change in the problem instance may affect both the distances between the cities and the number of cities (vertices). This figure presents an example of a DTSP that includes a primary sub-problem ($I_{0}$) and three successive sub-problems.

A simple example of a DOP is the dynamic traveling salesman problem (DTSP). This consists of a sequence of static traveling salesman problem (TSP) instances (sub-problems). Each successive sub-problem is created on the basis of the previous one. A portion of the sub-problem’s data is transferred unchanged from the predecessor, while the remaining portion is modified. Figure 1 summarizes this concept.

The aim is to find an optimal solution to every sub-problem. If all the sub-problems are solved to optimality, then the resulting sum of the solution (route) lengths is also minimal. Our DTSP benchmark generator includes the optimal value for every sub-problem; hence, the aim can be expressed in terms of finding the minimal sum of the differences between the generated solutions and the optima for the sub-problems. If the value of the sum is zero, then the optimum value is found for every sub-problem.

The particle swarm optimization (PSO) algorithm is an optimization technique created by Kennedy and Eberhart [3] in 1995. This technique is inspired by the natural behavior of a group of animals, e.g., a shoal of fish or a flock of birds. Every particle represents one of the possible solutions to a problem. In the continuous optimization case, the solution is a point in a real-valued space. The movement of the swarm can be interpreted as searching in the solution space. At the beginning of execution of the algorithm, the position of each particle is chosen randomly. Then, in each iteration of the algorithm, the velocities of the particles are calculated (direction of searching) and their positions updated. This results in a new solution to the problem. The velocity of the particle is influenced by the best-so-far solution (position) of the swarm and the best-so-far (previous) position of the particle. This process allows the swarm of particles to learn and move towards the areas of the problem solution space that contain higher quality solutions. The movement of the swarm in the solution space is described by the following equations:

(1) $$\begin{array}{cc} {\vec{v}}_{i}^{k+1}\leftarrow & \omega \cdot {\vec{v}}_{i}^{k}\\ & +\vec{U}\left(0,\phi_{1}\right)⊗\left({\vec{pBest}}_{i}-{\vec{x}}_{i}^{k}\right)\\ & +\vec{U}\left(0,\phi_{2}\right)⊗\left(\vec{gBest}-{\vec{x}}_{i}^{k}\right)\;, \end{array}$$

(2) $$\begin{array}{cc} {\vec{x}}_{i}^{k+1} & \leftarrow {\vec{x}}_{i}^{k}+{\vec{v}}_{i}^{k+1}\;,\\ & \vec{v},\;\vec{x},\;\vec{gBest},\;{\vec{pBest}}_{i}\in R^{n}\;, \end{array}$$

where *i* indexes the particles, *k* is the current iteration, ${\vec{v}}_{i}^{k}$ is the velocity of the *i*-th particle in the *k*-th iteration, ${\vec{x}}_{i}^{k}$ is the position of the particle equal to one of the solutions of the problem, the function U(0,ϕ) takes a uniform random value in the range [0,ϕ], and ω is an inertia parameter. The variables ${\vec{pBest}}_{i}$ and $\vec{gBest}$ denote the best-so-far solutions found by the particle and by the swarm, respectively, and $\phi_{1}$ and $\phi_{2}$ are cognitive and social parameters, respectively, that scale the influence of $\vec{pBest_{i}}$ and $\vec{gBest}$ on the position of the next particle.

Initially, the algorithm was created for optimization in a continuous space, but was later adapted to discrete optimization. In 1997, Kennedy and Eberhart [4] presented the first discrete PSO (DPSO) algorithm, in which the particle position was a binary vector and the velocity (direction of movement through the solution space) was the probability of a binary negation of the bits of the particle position. In 2004, Clerc [5] proposed a new DPSO algorithm and applied it to solve the TSP. In this algorithm, the particle position was a vector of vertices, while the velocity comprised a list of pairs of vertices, which changed in the next solution. In 2007, Shi et al. [6] presented an improved DPSO algorithm, which they used to solve the following TSP instances: *eil51*, *berlin52*, *st70*, *eil76*, and *pr70*. In their algorithm, the particle position is a permutation, and its modification resembles the well-known crossover found in genetic algorithms. Shi et al. showed that the proposed algorithm is capable of solving the generalized TSP, in which the edge lengths do not satisfy the triangle inequality. The homogeneous and heterogeneous versions of the DPSO algorithm described in the present paper are based on work by Zhong et al. [7] and on our previous DTSP variant [8]. In the implementation presented here, the particle position comprises a set of edges connecting TSP cities (nodes) and the corresponding probabilities of selecting the edges to the next solution (the next position of the particle). As far as we know, our previous work on applying the DPSO to solving the DTSP was the first publication on this topic in the literature.

The original PSO algorithm and its discrete versions are homogeneous, i.e., all particles have the same values of the parameters and hence share the same pattern of moving through a solution space [4]. However, heterogeneous populations are common in a natural environment [9]. One of the most important problems with the PSO concerns the balance between exploration and exploitation. A heterogeneous population allows particles to have various patterns of moving through the solution space and thus to exhibit different levels of emphasis on the exploitation and exploration of the solution space. It is possible that some of the parameter values might turn out to be useful at the beginning of the algorithm runtime, and others in the later stages. In this way, the balance between exploration and exploitation can be influenced [10,11].

### 1.1. Self-Adaptivity

To solve hard problems, algorithms like bio-inspired algorithms use different models, which change their behavior and allow performing specific tasks, like keeping a population diversity, increasing a search range, etc. Switching a model in order to solve a problem in a changing environment is called adaptation. Self-adaptivity can, therefore, be defined as the capability of a system to achieve its goals in a changing environment, by autonomously executing and switching between models [12]. Adaptivity has been widely studied since the mid-1960s, and several application areas relating to self-adaptivity have assumed greater importance. Thereafter, the scientific literature on self-adaptivity has been extensive, mainly over the past 16 years [13]. Self-adaptivity has been studied from the perspectives of software engineering, systems theory, artificial intelligence, and computer science, providing theoretical foundations and application fields such as: control engineering, mobile and autonomous robots, multi-agent systems, fault-tolerant computing, dependable computing, distributed systems, autonomous computing, self-managing systems, autonomous communications, adaptable user interfaces, machine learning, economic and financial systems, business and military strategic planning, sensor networks, pervasive and ubiquitous computing, etc. [13]. Another application is the self-adaptivity of bio-inspired algorithms like:

- evolution algorithms [14],
- differential evolution [15],
- particle swarm optimization [16].

Self-adaptivity from the perspectives of bio-inspired algorithms indicates automatic adjustment of an algorithm’s control parameters. Control parameters can be of various forms, for example: mutation rates, recombination probabilities, population size, or selection operators. We have combined particle swarm optimization with self-adaptation through the implementation of the characteristic sets of parameter values’ switching mechanism, as described in the Section 5.

### 1.2. Contributions

Our previous work focused mainly on DPSO with homogeneous (uniform) parameter values [8]. In this paper, we extend our initial work on DPSO in which individual particles may have non-uniform (varying) values of the parameters [17]. Specifically, our contributions are as follows:

- We propose a method for automatically setting the values of four crucial DPSO parameters. This method is based on discrete probability distributions defined to diversify the behaviors of the particles in the heterogeneous DPSO. The aim of this diversification is to improve the convergence of the algorithm.
- We perform an analysis of the convergence of the proposed algorithm based on computational experiments conducted on a set of DTSP instances of varying sizes. We discuss the relationships between the values of the DPSO parameters and their effect on particle movement through the problem’s solution search space.
- We study the diversity of the population of particles in the proposed heterogeneous DPSO and the original approach based on the information entropy calculated in two ways. The former method considers the edges, which are building blocks of the solutions to the TSP and DTSP. The latter focuses only on the quality of the solutions
- We compare the efficiency of the proposed heterogeneous DPSO with that of the base DPSO and two algorithms based on ant colony optimization (ACO). The results show that the proposed algorithm outperforms the base DPSO and is competitive with the ACO-based algorithms.

The structure of this paper is as follows. Section 2 presents a review of the literature concerning the DTSP. Section 3 describes the heterogeneous version of PSO. Section 4 gives a brief description of DPSO with pheromone. Section 5 describes the heterogeneous swarm. Section 6 presents our experimental results. Finally, Section 7 presents a summary and conclusions.

## 2. Dynamic Traveling Salesman Problem

The dynamic nature of the DTSP can entail changes in the distances between cities (nodes) and in the number of cities to be visited [18,19]. Every data transformation can trigger changes in local and global optima. The distance matrix can be defined as:

(3) $$D\left(t\right)={\left\{d_{ij}\left(t\right)\right\}}_{n(t)\times n(t)}\;,$$

where *t* is time, *i* and *j* denote vertices, and *n* is the number of vertices. Most often, it is assumed that the time is discrete, and hence, the DTSP can be viewed as a series of static TSP instances (Figure 1). Each sub-problem can be more or less similar to the previous one, depending on the number of changes and their magnitude. In this paper, we assume that only the distances between the cities are subject to change, while the number of nodes (vertices) remains constant.

Obviously, each of the DTSP sub-problems can be solved separately using one of the methods developed for the TSP [20]. Nevertheless, if the differences between consecutive DTSP sub-problems are small, it is possible that the optimal solutions differ only slightly. In such a case, it is possible to use the knowledge gathered while solving the previous sub-problem to speed up solving the current one. A summary of recent research on solving the DTSP that has been presented in the literature is given in Table 1.

## 3. Heterogeneity

Heterogeneity can be defined as the absence of uniformity (diversity). In computational intelligence algorithms, it can appear in many ways. A taxonomy of the various levels of heterogeneity that are possible in the PSO algorithm was given by Montes de Oca et al. [11], who divided heterogeneity into the following four categories:

1. *Neighborhood heterogeneity*: This concerns cases in which the size of the neighborhood is different for every particle, and hence, the virtual topology of connections between particles is not regular. Some particles can have a wider influence than others on the movement of the swarm.
2. *Best-particle heterogeneity*: Here, there can be variations in the method of selecting the best particle, i.e., the particle whose position is used when updating the current velocity and position. For instance, one particle might update its position following the best particle in its (small) neighborhood, while the second particle might be fully informed and follow the global best particle.
3. *Heterogeneity of the position update strategy*: Here, the particles differ in their patterns of movement (searching) through the solution space. For example, one group of particles might *explore* the solution space, while the other group might conduct a local search by restricting their velocities or even positions to a certain range. This type of heterogeneity diversifies the population to the greatest extent, since it provides the greatest flexibility in diversifying particle movement.
4. *Heterogeneity of parameter values*: Here, each particle or group of particles in the swarm can have different values of the parameters. For example, some particles might have a large inertia ω and explore the solution space, whereas other particles might have a small value of ω and perform the search locally (around the best position found). Although this type of heterogeneity is not as flexible as the heterogeneity of the position update strategy, it requires relatively few changes to the PSO, since only the values of the particle parameters need be set individually. It is this strategy that we apply in the proposed heterogeneous DPSO algorithm.

Although there is a lack of information in the literature with regard to heterogeneity in the case of the DPSO algorithm, it is possible to adapt the solutions proposed for standard PSO.

There are several methods of measuring population diversity in the population-based optimization algorithms. One of them is entropy, which is a formal method often found in the literature. Essentially, entropy is a measure of disorder or uncertainty. In information theory, it determines the amount of information within data and is frequently used for analytical purposes. It can also be used to control the behavior of an algorithm, e.g., as a stopping criterion [36,37] or a signal for resetting the population if the entropy drops below a threshold value [38]. It can be applied as a criterion for achieving a specified diversity of the initial population, as well. For example, new solutions can be created until the entropy exceeds a specific limit, which allows improving the convergence to the optimal solution [39,40].

The solution proposed in the article uses the last category of heterogeneity of the above classification: a variety of parameter values.

## 4. DPSO with Pheromone

A (homogeneous) DPSO algorithm with pheromone was proposed in our previous work [8], and this section contains only a brief description. Adaptation to a discrete space forces some changes to the original PSO algorithm designed for solving continuous optimization problems. All variables (i.e., *X* and *V*) become *sets* of edges instead of real-valued vectors. An edge is represented by a tuple: 〈p,{a,b}〉, where *a* and *b* are endpoints and *p* is the probability of selecting the edge (a,b) to become part of the constructed solution. The solution to the TSP problem is the set of edges that form the Hamilton cycle. The equations governing the movement of the particles become:

(4) $$\begin{array}{cc} V_{i}^{k+1}= & c_{2}\cdot U\left(0,1\right)\cdot \left(gBest\setminus X_{i}^{k}\right)\\ & \cup c_{1}\cdot U\left(0,1\right)\cdot \left(pBest_{i}\setminus X_{i}^{k}\right)\\ & \cup \omega \cdot V_{i}^{k}\;, \end{array}$$

(5) $$\begin{array}{cc} X_{i}^{k+1}= & \Delta \tau^{k}\left(V_{i}^{k+1}\right)⊕c_{3}\cdot U\left(0,1\right)\cdot X_{i}^{k}\;, \end{array}$$

where *i* is the particle index, *k* is the iteration, and U(0,1) is a uniform random number from the range [0,1]. The operators ∪ and ∖ denote the classical operations on sets, while the multiplication of a set by a scalar (i.e., $c_{2}\cdot U\left(0,1\right)\cdot \left(gBest\setminus X_{i}^{k}\right)$) represents multiplication of the *p* value of each edge by the scalar. The ⊕ operator does not exist in classical PSO; its purpose in DPSO is to complete the solution with missing edges so that it forms a Hamiltonian cycle. After the velocity is calculated, the result set may not create a Hamiltonian cycle. To create a feasible solution, the algorithm will be adding edges from nearest neighbor heuristics until a feasible solution is created. The Δτ function changes the probability *p* of the edge using the pheromone matrix familiar from ACO. The pheromone has two main functions in the algorithm:

1. It alters the probability of edge selection during the solution construction process; i.e., the higher the value of the pheromone, the greater is the probability of selecting the corresponding edge. In other words, the pheromone serves as an additional memory of the swarm, allowing it to learn the structure of high-quality solutions and, potentially, improve the convergence of the algorithm.
2. The pheromone matrix created while solving the current DTSP sub-problem is retained and used when solving the next sub-problem. This allows knowledge about the previous solution search space to be transferred with the aim of helping the construction of high-quality solutions to the current sub-problem. This implicitly assumes that the changes between consecutive sub-problems are not very great, so that the high-quality solutions to the current sub-problem share most of their structure with the high-quality solutions to the previous one.

For example, let $G_{u}$ be the undirected graph defined as follows:

Vu={1,2,3,4,5,6},|Eu|=n2

(all two-pair combinations of the set $G_{u}$). Let the first particle in the first iteration represent the solution:

$$\begin{array}{cc} X_{0}^{1} & =\{\langle 1,\{1,4\}\rangle ,\langle 1,\{4,2\}\rangle ,\langle 1,\{2,5\}\rangle ,\langle 1,\{5,3\}\rangle ,\langle 1,\{3,6\}\rangle ,\langle 1,\{6,1\}\rangle \}\;,\\ V_{0}^{1} & =\{\langle 1,\{2,5\}\rangle \},\\ gBest & =\{\langle 1,\{1,2\}\rangle ,\langle 1,\{2,3\}\rangle ,\langle 1,\{3,4\}\rangle ,\langle 1,\{4,5\}\rangle ,\langle 1,\{5,6\}\rangle ,\langle 1,\{6,1\}\rangle \}\;,\\ pBest_{0} & =\{\langle 1,\{1,2\}\rangle ,\langle 1,\{2,3\}\rangle ,\langle 1,\{3,5\}\rangle ,\langle 1,\{5,4\}\rangle ,\langle 1,\{4,6\}\rangle ,\langle 1,\{6,1\}\rangle \}\;. \end{array}$$

The result of applying Equation (4) is:

$$\begin{array}{cc} gBest\setminus X_{0}^{1} & =\{\langle 1,\{1,2\}\rangle ,\langle 1,\{2,3\}\rangle ,\langle 1,\{3,4\}\rangle ,\langle 1,\{4,5\}\rangle ,\langle 1,\{5,6\}\rangle \}\;,\\ pBest_{0}\setminus X_{0}^{1} & =\{\langle 1,\{1,2\}\rangle ,\langle 1,\{2,3\}\rangle ,\langle 1,\{5,4\}\rangle ,\langle 1,\{4,6\}\rangle \}\;. \end{array}$$

The next velocity of the particle $V_{0}^{2}$ after the operation of multiplication by $c_{1}\cdot rand\left(\right)$, $c_{2}\cdot rand\left(\right)$, or ω·rand() is:

$$\begin{array}{cc} \left(gBest\setminus X_{0}^{1}\right)\cup \left(pBest_{i}\setminus X_{0}^{1}\right)\cup V_{0}^{1}=\{ & \langle 0.3,\{1,2\}\rangle ,\langle 0.1,\{2,3\}\rangle ,\langle 0.5,\{3,4\}\rangle ,\langle 0.6,\{4,5\}\rangle ,\langle 0.1,\{5,6\}\rangle ,\\ & \langle 0.2,\{1,2\}\rangle ,\langle 0.9,\{2,3\}\rangle ,\langle 0.7,\{5,4\}\rangle ,\langle 0.4,\{4,6\}\rangle \}\;. \end{array}$$

The edge from the previous velocity is not added to the sum, because of the rule forbidding any vertex (node) from occurring more than four times (deg(2)=5) [7]. Let us assume that pheromone reinforcement is equal to zero (no influence) and that the random function returns the values 0.1, 0.7, 0.49, 0.5, 0.9, 0.3, 0.6, 0.55, 0.39. Then, after the filtration stage, the (incomplete) particle position set is:

$$X_{0}^{2}=\left\{\langle 0.3,\left\{1,2\right\}\rangle ,\langle 0.5,\left\{3,4\right\}\rangle ,\langle 0.6,\left\{4,5\right\}\rangle ,\langle 0.9,\left\{2,3\right\}\rangle ,\langle 0.7,\left\{5,4\right\}\rangle ,\langle 0.4,\left\{4,6\right\}\rangle \right\}\;.$$

The next stage is more restrictive. Any edge that creates an incorrect tour is removed from the set. The edge 〈0.4,{4,6}〉 is rejected, because deg(4)=3. The edge 〈0.7,{5,4}〉 is also rejected, because the edge with {4,5} endpoints already exists in the next position. The next incomplete particle position is:

$$X_{0}^{2}=\left\{\langle 0.3,\left\{1,2\right\}\rangle ,\langle 0.5,\left\{3,4\right\}\rangle ,\langle 0.6,\left\{4,5\right\}\rangle ,\langle 0.9,\left\{2,3\right\}\rangle \right\}\;.$$

At this stage, the first part of Equation (5) is completed. The operation ⊕ adds to the result the edge $\left(X_{0}^{2}\right)$〈1,{6,1}〉 chosen from the previous particle position set. To complete the set to form the Hamiltonian cycle, the nearest-neighbor heuristic is used, and the edge 〈1,{5,6}〉 is selected. The final particle position is:

$$X_{0}^{2}=\left\{\langle 1,\left\{1,2\right\}\rangle ,\langle 1,\left\{2,3\right\}\rangle ,\langle 1,\left\{3,4\right\}\rangle ,\langle 1,\left\{4,5\right\}\rangle ,\langle 1,\left\{5,6\right\}\rangle ,\langle 1,\left\{6,1\right\}\rangle \right\}\;.$$

**Figure 2:**
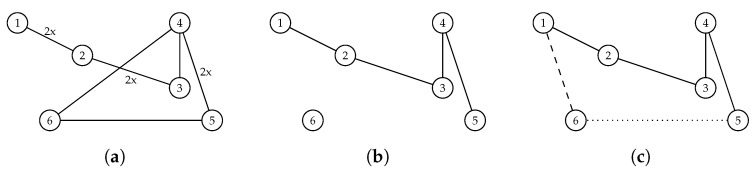
Example of the calculation of a new particle position in the DPSO, (**a**) before the filtration, (**b**) after the filtration, and (**c**) the final particle position.

Figure 2 presents a visualization of all the primary operations, i.e., the edges from the particle’s previous position $X_{i}^{k-1}$ before the filtration (a), after the filtration (b), and the final particle position (c). The dashed line marks the edge from Equation (5) and the dotted line the edge from the completion process (c).

## 5. Heterogeneous Swarm

The DPSO has four main parameters that influence particle movement through the solution search space: $c_{1}$, $c_{2}$, $c_{3}$, and ω. To better understand how the parameter values are set in the proposed *heterogeneous* DPSO, it is helpful to focus on how the parameters govern the swarm behavior. Zhong et al. [7] suggested the following ranges of values for the parameters: $c_{1}\in \left[0,1.5\right]$, $c_{2},c_{3}\in \left[0,2\right]$, ω∈[0,0.6]. Setting the parameters to small values, i.e., close to the start of the range, forces the particles to change their positions (edges) frequently, since the probability of selecting the edges from the current best positions (local and global) is relatively small. Furthermore, in the initial stage of execution of the algorithm, the pheromone values cannot guide the construction process, since they are also small. On the other hand, setting the parameters to higher values forces the solution construction process to become more exploitative, since the constructed solutions resemble the previously-obtained high-quality solutions. Based on the expertise gathered during our earlier studies of the DPSO algorithm, we have selected the *characteristic sets* of the parameter values, which are shown in Table 2. For each set, we provide a brief description of the corresponding DPSO particle behavior. Below, we present a more detailed description of the sets, supported by some experimental data analysis.

**Figure 3:**
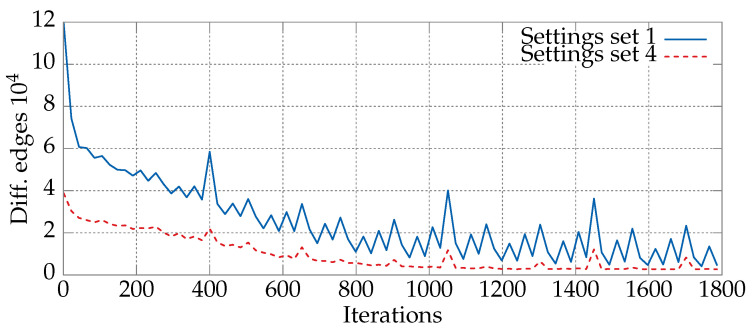
Numbers of new (different) edges between $X^{k-1}$ and $X^{k}$ (the previous and current positions) in the DPSO solving the static *kroA200* TSP instance (200 cities). The blue line indicates the particles with the first set of values from Table 2 (Setting 1), and the red line is for the more “stable” particles for which the fourth set (Setting 4) of parameter values was used. The remaining parameters were taken from Table 3. The values were averaged over 30 runs of the algorithm.

Figure 3 presents the numbers of new edges for $X^{k-1}$ and $X^{k}$ (the previous and current positions). The blue line indicates the particle parameter values, which often change edges (Setting 1), and the red line is for more stable particles, with less frequent changes (Setting 4).

The first and fourth sets of parameter values from Table 2 differ in terms of the dynamics of changes in the number of common edges between the current and previous positions of the particle. For the small parameter values taken from the first set, the probability of edge selection to the next position (*p*) is very small and can only be increased if the corresponding pheromone has a high value. On the other hand, in the fourth set of parameters, $c_{3}$ has the highest value from the range. As a result, the edges from the previous position will be added to the next position of the particle with high probability. Both characteristics can be clearly seen in Figure 3. The blue line is below the red one, which means that the position of the particle from the first set has more changed edges.

**Figure 4:**
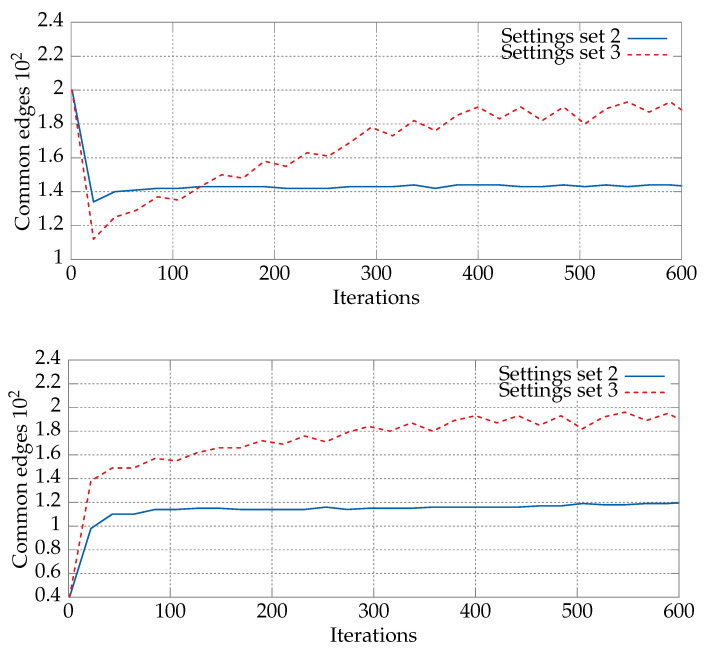
Numbers of common edges between $X^{k}$ and pBest (top) and $X^{k}$ and gBest (bottom) for the second and third sets of characteristic parameter values (Table 2). The DPSO algorithm was run for the static *kroA200* TSP instance (200 cities). The remaining parameters were taken from Table 3. The values were averaged over 30 runs of the algorithm.

An analogous comparison can be made for the second and third sets of values shown in Table 2. Figure 4 shows the average numbers of common edges between the current position of a particle, $X^{k}$, and the best positions, i.e., the particle’s local best pBest and the swarm’s best gBest. For the second set of parameter values, the number of edges shared with pBest was higher than for the third set. This was caused by the high $c_{1}$ value, equal to two, which affected in particular the initial iterations of the algorithm. After the first 100 iterations, the number began to change as pBest and gBest became more similar. This is an effect of the high value of the $c_{2}$ parameter in the third set of parameter values. The bottom plot in Figure 4 shows the average number of common edges for the sets $X^{k}$ and gBest. We can see a growing similarity of the current position $X_{k}$ to the current best position pBest. This effect can be observed for both sets of parameter values. The number of common edges was higher for the third set, since it had the highest possible value of $c_{1}$.

**Figure 5:**
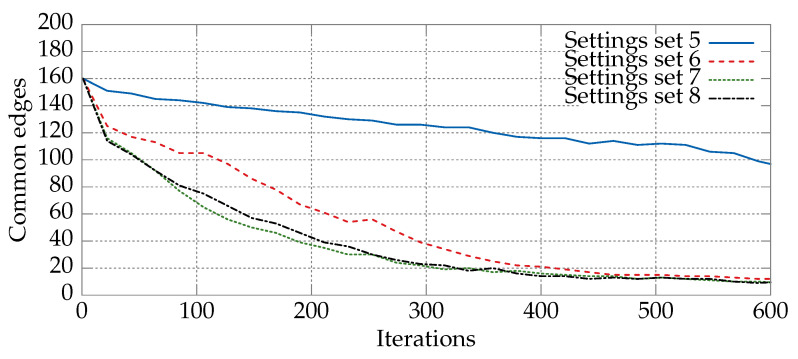
Total numbers of common edges between the current position of the particle, $X^{k}$, and pBest, and between $X^{k}$ and gBest for the DPSO solving the *kroA200* TSP instance (200 cities) with the parameters given by Sets 5–8 in Table 2. The remaining parameters were taken from Table 3. The values were averaged over 30 runs of the algorithm.

An analogous comparison, this time for Sets 5–8 from Table 2, is presented in Figure 5. The largest differences can be observed for the fifth and the sixth sets, and the smallest for the seventh and eighth. This is due mainly to the small differences between the parameter values, namely $\Delta c_{1}=0.25$ and Δω=0.25 (the remaining parameters $c_{2}$ and $c_{3}$ have the same value).

Based on the number of times each value of a parameter appears in Table 2, an independent discrete probability distribution for the parameters can be defined:

1. $c_{1}$: P(0.1) = 0.4, P(0.75) = 0.15, P(1.5) = 0.3, P(1.75) = 0.15;
2. $c_{2}$ and $c_{3}$: P(0.1) = 0.4, P(1) = 0.15, P(1.5) = 0.15, P(2) = 0.3;
3. ω: P(0.1) = 0.4, P(0.25) = 0.2, P(0.5) = 0.4.

This allows the values of the DPSO parameters to be controlled, while also allowing them to be mixed together; i.e., any combination of the listed values is possible. As a result, we can expect that both the exploration- and exploitation-oriented behaviors of the particles will be present in a swarm, hence increasing the chances of finding high-quality solutions regardless of the “landscape” of the solution space. This also has the advantage of being more computationally efficient compared with a completely-random setting (e.g., with uniform probability), since, in the latter case, one would need a larger number of particles to observe a similar mix of characteristic particle behaviors.

## 6. Experimental Results

This section is divided into three parts. In the first, we focus on the effect of the parameter values on the performance of individual particles in the heterogeneous DPSO algorithm. In the second, we conduct a comparison between the homogeneous DPSO, the proposed heterogeneous DPSO, and two well-known ACO algorithms, namely the ant colony system (ACS) and population-based ACO (PACO). The third part presents a study of the population entropy and its influence on the algorithm convergence.

### 6.1. Convergence Analysis for Various Sets of Parameters

To assess the performance of individual particles in a swarm of the heterogeneous DPSO, we counted the number of times the particle improved the current global best solution gBest. The parameter values were set randomly according to the discrete probability distribution described in Section 5, independently of the other parameters values. The *gr666* TSP instance (666 cities) was used as a test bed.

Table 4 shows the sets of parameter values for which the particles were able to improve the global best solution most frequently. As can be seen, the top two are the sets in which the parameters $c_{1}$, $c_{2}$, $c_{3}$, and ω are relatively small. These values favor exploratory behavior of the DPSO particles, and hence, the particles are more likely to find an improved solution, especially in the initial phases of algorithm execution. The set for which the behavior should be more stable and less exploratory, i.e., with $c_{2}=2$, turned up as third in the ranking. The relatively large difference of 53 between the second and third positions is also noteworthy. The lower rankings of the particles exhibiting more exploitative behavior confirmed that they could be more important in the later stages of algorithm execution, in which smaller changes to the solution structure are preferred.

**Figure 6:**
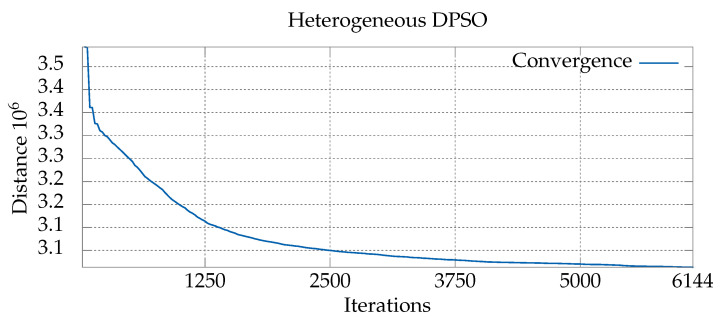
Chart showing convergence with the optimum of the heterogeneous version of the algorithm for the *gr666* problem.

To clarify this distinction, we analyzed which values of the parameters proved to be working best during subsequent phases of algorithm execution. The phases were defined by dividing the total number of iterations into equal parts (intervals). For each interval, we ranked the sets of parameter values based on the number of times they led to a new global best solution within the respective interval. Table 5 presents the results, while Figure 6 shows the speed of convergence towards an optimum in each phase. As can be seen, different sets of parameter values dominate subsequent phases (intervals) of the computations. In the first interval (0–1250), the sets with small parameter values are predominant, which indicates that rapid changes in the particle solutions are beneficial. In the third interval (2500–3750), the sets of parameter values are mixed, i.e., they contain both small and high values. This can be interpreted as a sign that the exploration of the solution space slows down and, more importantly, becomes exploitation. In the last interval (5000–6144), the best particles have relatively high parameter values, which, combined with stronger pheromone reinforcement, causes mainly small changes to the particle positions.

### 6.2. Comparative Study

To evaluate the performance of the proposed DPSO algorithm, we compared it with the homogeneous version of the DPSO and with ACS and PACO, which are among the best-performing metaheuristics for the TSP and DTSP problems. The DTSP test instances were generated based on the static TSP instances from the well-known TSPLIB repository. The test data can be found in a public repository (https://github.com/lukaszstrak/DTSP-repository). Algorithm 1 presents an outline of the general test procedure used to solve the DPSO with the algorithms mentioned.

**Algorithm 1** Outline of the procedure for solving the DTSP.
Load the static TSP instance  ▹ The original TSP instance becomes the first DTSP sub-problemInitialize the algorithm-related data**while** Stop criterion is not met **do**    sub-problem-related initialization            ▹ Create swarm, neighborhood, etc.    Solve the current sub-problem                ▹ Solve with DPSO, ACO, etc.    Modify the current sub-problem to obtain the next one
**end while**



**Figure 7:**
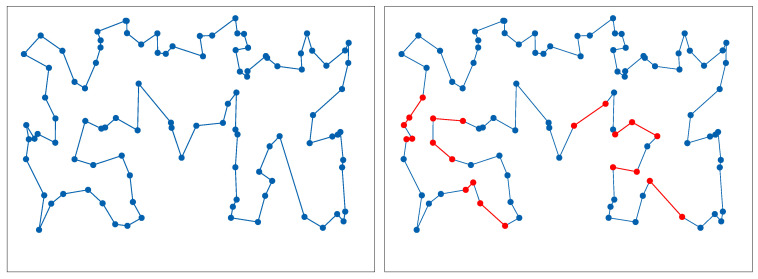
Visualization of the optimum routes for the static *kroA100* TSP instance (left side) and the DTSP instance after a random relocation of some cities (right side). The edges differentiating the new optimum from the previous are marked in red.

To make the comparison fair, all algorithms were solving the same DTSP instances, i.e., starting from the same static TSP and including the same DTSP-related changes to the positions of the cities. Each DTSP instance comprised 11 static TSP sub-problems, namely the original problem from TSPLIB and ten sub-problems resulting from random changes to the position of the cities. The *gr666* problem was an exception, since it included only one sub-problem (the original TSPLIB problem). Figure 7 shows an example of a DTSP instance consisting of two static TSP sub-problems. The optimum solutions for each of the DTSP sub-problems were obtained using the well-known Concorde solver by Applegate et al. [41]. The number of changes in the city position between successive sub-problems was set to 3% in all DTSP cases.

Table 6 shows the parameter values of the two DPSO variants. The numbers of iterations used are shown alongside the results in Table 7. The size of the swarm and the size of the particle neighborhood were determined from preliminary computations, keeping in mind that both parameters strongly influence the computation time and the quality of the solutions. A smaller neighborhood limits the solution space and speeds up computation. However, too low a value could hamper finding the optimum. The parameters ($c_{1}$,$c_{2}$, $c_{3}$, ω, SwarmSize, and neighborhood) for the *homogeneous* version of the DPSO were chosen based on preliminary computations and our previous work on DPSO.

The ACS and PACO parameters were set as follows: number of ants = 10; number of iterations = $\left\lfloor 0.1\cdot p_{ev}\right\rfloor$; β=3; local and global pheromone evaporation coefficients α=0.1 and ρ=0.1, respectively; and $q_{0}=\left(n-10\right)/n$, where *n* is the size of the problem. For the PACO algorithm, $q_{0}=0.8$ was used, and the *age-based* strategy for updating the solution archive (of size five) was used. The values of the parameters were set based on preliminary computations and the suggestions by Cáceres el al. [42], in which the ACO was tested with a small computation budget.

All the considered algorithms, including DPSO and ACO, were allowed to construct and evaluate exactly the same number of solutions ($p_{ev}$) to a problem. For example, the DPSO algorithm with 104 iterations and a swarm of size 32 constructed a total of $p_{ev}=104\cdot 32=3328$ solutions. All algorithms were implemented in the C# language and run on a computer with an Intel i7 3.2-GHz CPU. All computations were repeated 30 times, and the results were averaged.

For the smallest DTSP instance (*berlin52*), both DPSO versions generated results that were of similar quality and, at the same time, better than those of the ACO algorithms. For the larger instances, the heterogeneous DPSO showed a clear advantage over the homogeneous DPSO. The biggest differences were observed for the *pcb442* and *gr202* instances, for which the heterogeneous version generated higher quality solutions, especially if the number of iterations was low. This confirms that the heterogeneity of the parameter values resulted in a broader exploration of the solution search space. At the same time, the heterogeneous DPSO was also more consistent in finding high-quality solutions, which was manifested in the smaller average standard deviation compared with the homogeneous version. When the number of iterations grew, the advantage of the heterogeneous DPSO became less, confirming that, in the later stages of the computations, the exploitative nature of the algorithm became more important. Generally, both DPSO versions benefited from a larger number of iterations. Compared with the ACO algorithms, the results of the DPSO were worse in four out of six cases for the lowest number of iterations. However, for the largest number of iterations allowed, the DPSO lost only once (for the *kroA200* DTSP instance) even though the ACO algorithms also benefited from the larger computation time. This suggests that, compared with the ACO algorithms, the DPSO variants converged more rapidly, although their search through the solution space was more explorative at the beginning. Increasing the number of iterations past a certain point allowed DPSO to outperform ACS and PACO in almost all cases.

### 6.3. Entropy Study

**Figure 8:**
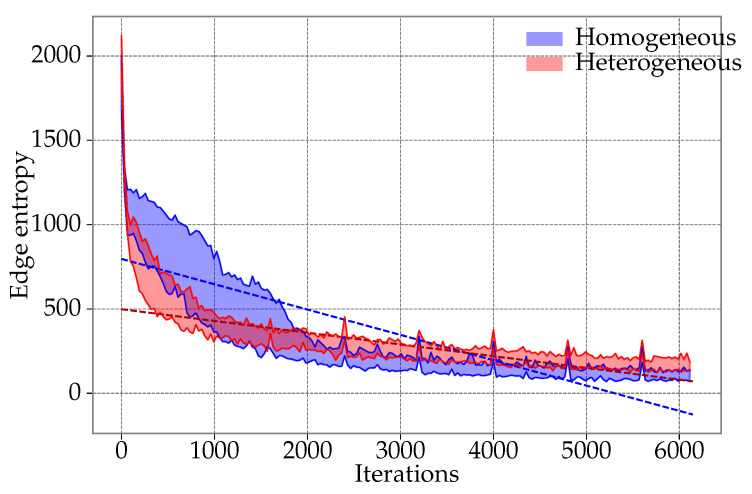
The comparison of the entropy of the homogeneous and the proposed heterogeneous DPSO for the *gr666* TSP problem instance. The entropy was calculated based on the numbers of occurrences of the edges comprising the particles’ solutions. The plot shows the spread of the entropy levels, which were measured in 30 executions of the algorithms.

Maintaining a diversified population during the execution of the DPSO algorithm is a desired feature that may reduce the chance of getting stuck in local minima. In this section, we study the diversity of the population in the proposed self-adaptive DPSO and the original DPSO approach. In order to estimate the diversification, we used the entropy, which is measured in two ways. In the first way, the measurement concerns the edges comprising the solutions from the population (particles). Specifically, we defined a discrete probability distribution by counting edges’ occurrences in the population. For example, in the two following solutions (particles’ positions): {{1,2},{2,3},{3,4},{4,1}}, and {{1,2},{2,4},{4,3},{3,1}}, the edges {1,2} and {3,4} appear two times and {2,3}, {1,4}, {2,4}, and {1,3} appear once; hence, the entropy equals: H=−2·22log2(22)+4·12log2(12)=2. The second measure of the entropy was defined analogously, but the length of the solutions was used in the place of the edges. Figure 8 shows the population entropy, which counts the number of different edges for the proposed heterogeneous DPSO and the original homogeneous DPSO. The results were obtained for 30 runs of the algorithms, which solved the *gr666* TSP problem instance. As can be seen, the entropy for the homogeneous DPSO was relatively large during the first few hundred iterations, but quickly fell to much lower levels. On the contrary, the entropy in the heterogeneous DPSO dropped faster during the initial stage of the algorithm execution, but after the first 2000 iterations, it consistently remained higher than in the homogeneous DPSO. The more balanced exploration to exploitation ratio resulting from the diversity of the particles’ behaviors can explain the higher levels of the entropy for the heterogeneous DPSO.

**Figure 9:**
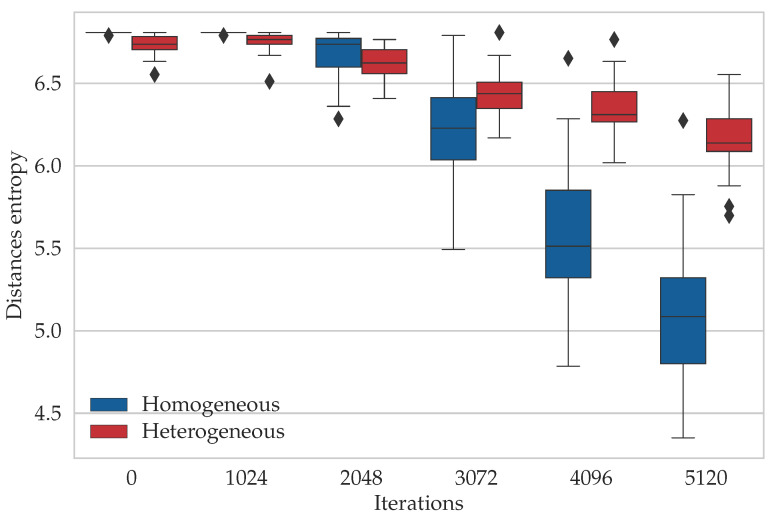
The comparison of the entropy, which was calculated based on the solution length in the populations of the homogeneous DPSO and the proposed heterogeneous DPSO for the *gr666* TSP problem instance. The plot shows the spread of the entropy levels, which were measured in 30 executions of the algorithms.

Figure 9 presents the box-plot of the entropy levels’ measurements based on the lengths of the solutions corresponding to the DPSO population. The results were consistent with the previous entropy observations, i.e., the solutions generated by the homogeneous DPSO were less diverse in terms of the tours lengths than the solutions obtained for the heterogeneous DPSO.

**Figure A1:**
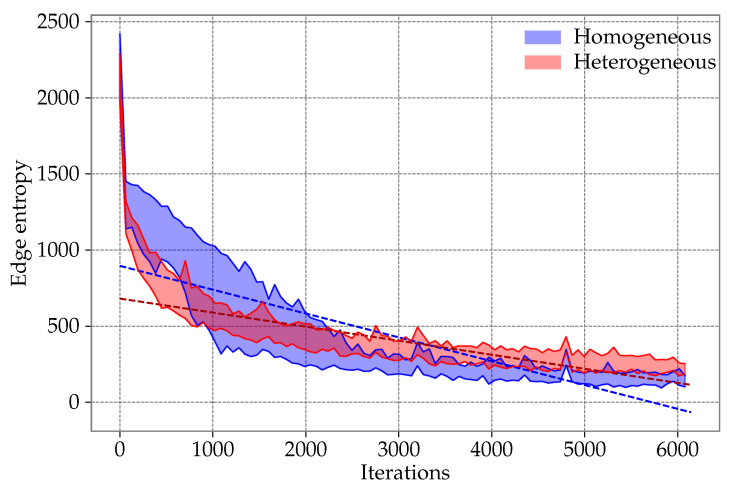
The comparison of the entropy of the homogeneous and the proposed heterogeneous DPSO for the *rat783* TSP problem instance. The entropy was calculated based on the numbers of occurrences of the edges comprising the particles’ solutions. The plot shows the spread of the entropy levels, which were measured in 30 executions of the algorithms.

**Figure A2:**
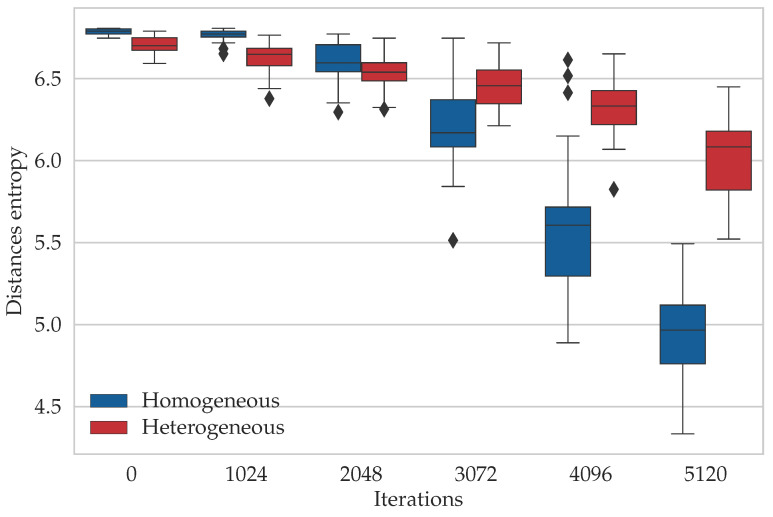
The comparison of the entropy, which was calculated based on the solution lengths in the populations of the homogeneous DPSO and the proposed heterogeneous DPSO for the *rat783* TSP problem instance. The plot shows the spread of the entropy levels, which were measured in 30 executions of the algorithms.

**Figure A3:**
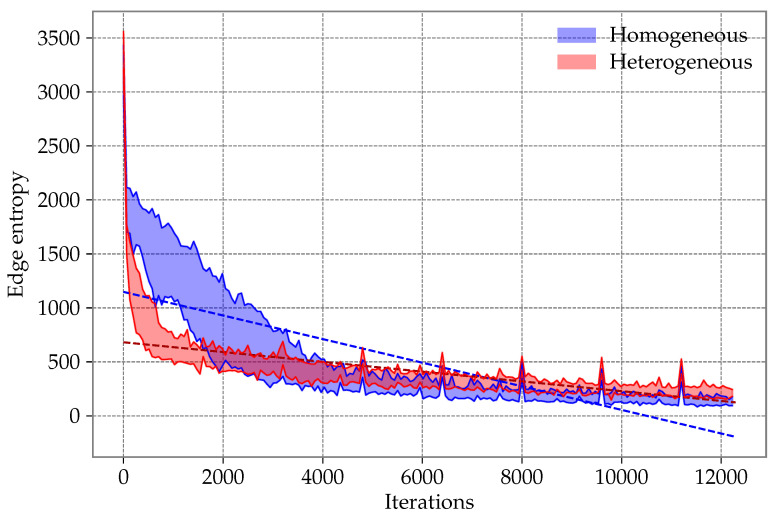
The comparison of the entropy of the homogeneous and the proposed heterogeneous DPSO for the *pcb1173* TSP problem instance. The entropy was calculated based on the numbers of occurrences of the edges comprising the particles’ solutions. The plot shows the spread of the entropy levels, which were measured in 30 executions of the algorithms.

**Figure A4:**
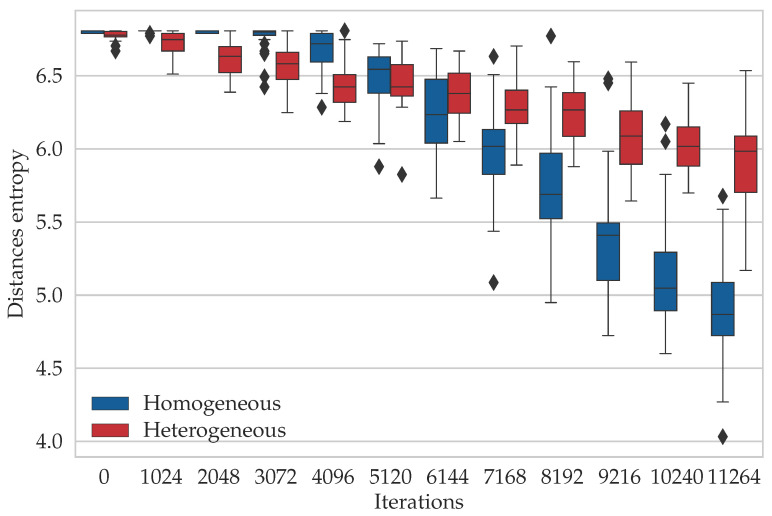
The comparison of the entropy, which was calculated based on the solutions lengths in the populations of the homogeneous DPSO and the proposed heterogeneous DPSO for the *pcb1173* TSP problem instance. The plot shows the spread of the entropy levels, which were measured in 30 executions of the algorithms.

Similar results were obtained for the *rat783* TSP instance (Figure A1 and Figure A2) and the *pcb1173* TSP problem instance (Figure A3 and Figure A4), which can be found in the Appendix section. The parameters values were used the same as for the *gr666* TSP problem instance (Table 6).

Summarizing, the presented study of the entropy confirmed the efficiency of the proposed method in enforcing the diversity of the behaviors of the particles in the DPSO. This was especially important in the later stages of the algorithm execution as the more diverse population increased the probability of escaping from local minima and often resulted in a higher quality of the final solutions, as confirmed by the results summarized in Table 7.

## 7. Conclusions

We have proposed a heterogeneous DPSO algorithm for solving the DTSP. In this algorithm, each particle can have different values of the crucial DPSO parameters $c_{1},c_{2},c_{3}$, and ω. These values were chosen randomly according to the discrete probability distribution defined so that different behaviors of the DPSO particles could be obtained. Computational experiments conducted on a set of DTSP instances showed that it is beneficial if some particles explore the solution space while others are more exploitative, i.e., narrow their search by constructing solutions similar to the high-quality solutions found so far. The diversity of the parameter values in the heterogeneous DPSO produced a higher entropy of the population of the generated solutions in comparison with the homogeneous variant of the algorithm. As a consequence, the heterogeneous DPSO algorithm improved the quality of the results obtained compared with the homogeneous version. Moreover, the algorithm was easier to use, since fewer parameters had to be set manually, which is important because choosing the right values of the parameters can be especially difficult for the DTSP. It is also worth emphasizing that both versions of the DPSO algorithm were comparable to the proven ACS and PACO metaheuristics in terms of solution quality. In fact, heterogeneous DPSO was able to generate solutions of better quality than both of the ACO-based algorithms in most cases, while also exhibiting more rapid convergence if the computation time was extended.

In the future, we plan to test different types of heterogeneity in addition to the parameter diversity considered here.

## Figures and Tables

**Table 1:** Summary of recent papers on solving the DTSP with computational intelligence methods.

Year	Authors	Algorithm	DTSP Variant
2001	Guntsch and Middendorf [21]	ACO with local and global reset of the pheromone	Addition/removal of vertices
2002	Eyckelhof and Snoek [22]	ACO with various variants of pheromone matrix update to maintain diversity	Changes in edge lengths with time (simulated traffic jam on a road)
2006	Li et al. [19]	GSInver-over and gene pool with the α-measure [23]	CHN145 + 1: 145 cities and one satellite
2010	Mavrovouniotis and Yang [24]	ACO with immigrants scheme to increase population diversity	Coefficients: frequency and size of changes
2011	Simões and Costa [25]	CHCalgorithm	A test involving the addition of changes and their subsequent withdrawal [26]. In that way, the optima at the beginning and the end are the same.
2014	Tinós et al. [27]	EA algorithm	Random changes in the problem
2014	Zhang and Zhao [28]	Hopfield neural network	Simulation of various types of real random events in a street
2016	Eaton et al. [29]	ACO with immigrants scheme	Changes in edge lengths. Simulated delays of trains.
2016	Mavrovouniotis and Yang [30]	MMAS	Encoding of the problem is changed, but the optimal solution remains the same
2017	Mavrovouniotis et al. [31]	ACO	Distances between cities are changed. The problem can be transformed to an asymmetric one.
2018	Chowdhury et al. [32]	ACO	Random DTSP, dynamic changes occur randomly. Cyclic DTSP, dynamic changes occur with a cyclic pattern.
2018	Schmitt et al. [33]	MMAS	Acyclic DTSP with changes in edge lengths with time
2018	Yirui Wang et al. [34]	ACO	
2018	Yan-Wei Huang et al. [35]	MCTS	Addition/removal of vertices

**Table 2:** Characteristic sets of particle parameter values for the DPSO algorithm along with their influence on particle movement.

No.	$c_{1}$	$c_{2}$	$c_{3}$	ω	Description
1	0.1	0.1	0.1	0.1	Favors quick changes of position
2	2.0	0.1	0.1	0.1	Emphasis on the information from pBest
3	0.1	2.0	0.1	0.1	Emphasis on the information from gBest
4	0.1	0.1	2.0	0.5	Very slow changes of position
5	0.75	1.0	1.0	0.25	Weak pBest, gBest influence
6	1.25	1.5	1.5	0.5	Stronger pBest, gBest influence
7	1.5	2.0	2.0	0.5	Strong pBest, gBest influence
8	1.75	2.0	2.0	0.75	Very strong pBest, gBest influence

**Table 3:** Values of DPSO-related parameters.

Problem	$c_{1}$	$c_{2}$	$c_{3}$	ω	SwarmSize	Neighborhood
*berlin52*	0.5	0.5	0.5	0.2	32	7
*kroA100*	0.5	0.5	0.5	0.5	64	7
*kroA200*	0.5	0.5	0.5	0.5	80	7
*gr202*	0.5	0.5	0.5	0.5	101	10
*pcb442*	0.5	1.5	0.5	0.5	104	15
*gr666*	0.5	1.0	1.5	0.6	112	30

**Table 4:** Ranking of parameter values after 6144 iterations for which the particles in the heterogeneous DPSO were able to improve the global best solution the greatest number of times. The results are accumulated over 30 executions for the *gr666* TSP instance.

Rank	Parameters				Number of gBest Improvements
	$c_{1}$	$c_{2}$	$c_{3}$	ω	
1	0.1	0.1	0.1	0.5	113
2	0.1	0.1	0.1	0.1	102
3	0.1	2	0.1	0.1	49
4	0.1	2	2	0.5	46
5	0.1	2	2	0.1	42
6	0.1	1.5	0.1	0.5	39
7	0.1	1	0.1	0.1	38
8	0.1	1	0.1	0.25	34
9	0.75	2	2	0.25	27
10	0.1	1	2	0.1	26
11	0.1	2	0.1	0.25	24
12	0.75	2	2	0.1	22
13	1.5	1.5	2	0.5	21
14	1.5	2	0.1	0.1	21
15	1.5	2	0.1	0.25	20

**Table 5:** Ranking of parameter values for which the particles in the heterogeneous DPSO were able to improve the global best solution the greatest number of times within five designed subsequent phases of the computations. The results are accumulated over 30 executions for the *gr666* TSP instance.

Iterations	Parameters				Number of gBest Improvements
	$c_{1}$	$c_{2}$	$c_{3}$	ω	
0–1250	0.1	0.1	0.1	0.1	94
	0.1	0.1	0.1	0.5	93
	0.1	2	2	0.5	38
	0.1	2	0.1	0.1	38
	0.1	2	2	0.1	32
1250–2500	0.75	2	2	0.25	12
	1.5	2	0.1	0.1	10
	0.1	2	0.1	0.1	10
	0.1	1.5	0.1	0.5	9
	0.1	1	2	0.1	8
2500–3750	0.1	0.1	0.1	0.5	10
	1.5	1.5	2	0.5	4
	1.5	2	0.1	0.25	4
	0.75	2	2	0.25	3
	0.1	1	2	0.1	3
3750–5000	0.1	1	0.1	0.1	2
	0.1	1	2	0.1	2
	0.75	0.1	2	0.5	2
	1.5	0.1	2	0.1	2
	1.5	2	1.5	0.1	2
5000–6144	1.75	2	1	0.5	3
	1.5	2	1.5	0.1	2
	1.75	0.1	2	0.5	2
	0.1	1.5	0.1	0.5	2
	1.5	1	1	0.1	1

**Table 6:** Values of DPSO-related parameters.

Homogeneous DPSO					Heterogeneous DPSO					Common Parameters	
**Problem**	$c_{1}$	$c_{2}$	$c_{3}$	ω	**Problem**	$c_{1}$	$c_{2}$	$c_{3}$	ω	SwarmSize	**Neighborhood**
*berlin52*	0.5	0.5	0.5	0.2	*berlin52*	Chosen randomly as described in Section 5				32	7
*kroA100*	0.5	0.5	0.5	0.5	*kroA100*					64	7
*kroA200*	0.5	0.5	0.5	0.5	*kroA200*					80	7
*gr202*	0.5	0.5	0.5	0.5	*gr202*					101	10
*pcb442*	0.5	1.5	0.5	0.5	*pcb442*					104	15
*gr666*	0.5	1.0	1.5	0.6	*gr666*					112	30

**Table 7:** Comparison of results for the homo and heterogeneous DPSO variants and the ACO algorithms obtained for four DTSP (*berlin52, …, pcb442*) and one TSP (*gr666*) instances. “G” denotes the distance to the optimum and “D” the average standard deviation of this distance. The numbers of iterations are given per sub-problem. The best solutions found by the DPSO algorithms are marked in boldface. All computations were repeated 30 times. PACO, population-based ACO.

Problem	Iterations	DPSO Algorithms						Counterparts	
		Homogeneous			Heterogeneous			ACS	PACO
		T (s)	G (%)	D (%)	T (s)	G (%)	D (%)	G (%)	G (%)
*berlin52*	104	0.13	0.15	0.32	0.13	**0.13**	0.15	0.96	0.96
*berlin52*	416	0.3	0.01	0.04	0.28	0.01	0.05	0.5	0.5
*berlin52*	1664	0.98	**0**	0	0.89	0.01	0.05	0.46	0.46
*kroA100*	100	1.03	5.44	2.47	0.86	**2.68**	1.4	1.8	2.97
*kroA100*	400	1.63	1.28	1.02	1.27	**1.05**	0.81	1.31	2.13
*kroA100*	1600	4.11	**0.64**	0.69	3.38	0.78	0.77	0.82	1.36
*kroA200*	160	2.49	15.63	2.77	2.18	**5.14**	1.84	2.41	3.33
*kroA200*	640	5.13	4.45	1.62	4.46	**2.89**	1.09	1.62	2.71
*kroA200*	2560	15.6	**1.62**	0.81	13.18	2.02	0.8	1.47	2.28
*gr202*	128	8.82	13.75	2.06	8.17	**4.19**	1.2	6.26	4.91
*gr202*	512	11.54	6.81	2.11	10.88	**1.97**	0.66	4.88	3.9
*gr202*	2048	23.01	**1.52**	0.6	21.98	1.53	0.55	3.93	3.34
*pcb442*	272	11.22	29.31	5.33	11.16	**6.73**	1.68	6.18	4.44
*pcb442*	1088	28.52	13.41	5	30.69	**2.87**	0.89	4.87	3.56
*pcb442*	4352	102.78	3.13	1.52	108.25	**1.92**	0.79	3.91	3.3
*gr666*	384	85.19	10.84	1.52	91.83	**9.58**	0.86	9.18	5.89
*gr666*	768	98.36	7.37	1.0	115.19	**6.88**	0.78	7.46	4.77
*gr666*	1536	124.84	5.62	0.84	163.48	**5.33**	0.57	6.09	4.51
*gr666*	3072	180.66	4.88	0.63	259	**4.52**	0.88	5.67	4.14
*gr666*	6144	296.83	3.99	0.77	453.83	**3.8**	0.78	4.92	4.21

## Abbreviations

The following abbreviations are used in this paper:

ACO	ant colony optimization
DPSO	discrete particle swarm optimization
DTSP	dynamic traveling salesman problem
PACO	population ant colony optimization
PSO	particle swarm optimization
TSP	traveling salesman problem
